# Menstrual Derangement

**Published:** 1890-07

**Authors:** D. D. Quillian

**Affiliations:** Athens, Ga.


### REPORTS OF CASES.

### MENSTRUAL DERANGEMENT.

I report the following case, thinking it may be of some interest to the profession :

Was called to see Mrs. W., aged 20, April 23d, at 6 o'clock A. M. Menstrual flow began afternoon preceding. She was attacked with dysentery after midnight. I found her pulse 120, temperature, 102½, menstrual flow stopped, considerable pain in lower abdominal region, very nervous. I ordered an opiate, also hot fomentations. Was called again at 8 o'clock; found pulse 140, temperature, 104½, patient delirious, with symptoms of convulsions. Gave full dose bromide pot. and chloral hyd., Norwood's Tinc. Verat. Vir. Sponged freely with ice water. At 9 o'clock temperature, 106; pulse, 150. Continued veratrum and sponging. At 10 o'clock temperature reduced to 105; pulse, 150. Gave eight grain doses of antipyrin which was vomited immediately. At 12:30 P. M. temperature reduced to 102½; pulse, 130. Saw patient again at 2 P. M.; temperature, 104½; pulse about 140. Gave antipyrin which was retained. At 6 P. M. temperature, 102½; patient resting quietly. Saw patient morning 24th; temperature normal; pulse, 100. For several days following temperature ranged from one degree above to one degree below normal. Menstrual flow returned day following and ceased fourth day after the attack and returned again the seventh day. Patient suffered great deal from uterine pains and nausea during the first week and made a very slow recovery.

D. D. QUILLIAN, M. D.,
Athens, Ga.

